# Genome Sequences and Metagenome-Assembled Genome Sequences of Microbial Communities Enriched on Phytoplankton Exometabolites

**DOI:** 10.1128/MRA.00724-20

**Published:** 2020-07-23

**Authors:** He Fu, Christa B. Smith, Shalabh Sharma, Mary Ann Moran

**Affiliations:** a Department of Marine Sciences, University of Georgia, Athens, Georgia, USA; University of Southern California

## Abstract

We report 11 bacterial draft genome sequences and 38 metagenome-assembled genomes (MAGs) from marine phytoplankton exometabolite enrichments. The genomes and MAGs represent marine bacteria adapted to the metabolite environment of phycospheres, organic matter-rich regions surrounding phytoplankton cells, and are useful for exploring functional and taxonomic attributes of phytoplankton-associated bacterial communities.

## ANNOUNCEMENT

Bacteria associated with marine phytoplankton play essential roles in global carbon and nutrient cycles (1, 2). In the nutrient-rich phycosphere surrounding phytoplankton cells, the interactions among cooccurring bacteria and between bacteria and their hosts determine community composition. The phycosphere therefore represents a unique microcosm in which ecological principles of microbial community assembly can be examined (3, 4). A high-throughput synthetic phycosphere system was established (5) in which a natural coastal seawater bacterial assemblage was passaged through 8 serial transfers in a 96-well format culture system containing different mixtures of metabolites known to be released preferentially by diatoms (6, 7) or dinoflagellates (7). Here, we report the genomes and metagenome-assembled genomes (MAGs) enriched in those synthetic phycospheres, providing a resource for understanding the functional potentials of, and ecological interactions occurring in, ocean microbial communities.

The 11 bacterial strains were isolated from 2 microbial communities, 1 supplied with a mixture of 5 diatom exometabolites (xylose, glutamate, glycolate, ectoine, and dihydroxypropanesulfonate) and the other with 5 dinoflagellate exometabolites (ribose, spermidine, trimethylamine, isethionate, and dimethylsulfoniopropionate) (5). Enrichments were serially diluted, plated onto solid marine basal medium containing each of the individual metabolites, and grown at 24°C in the dark for 1 week. Single colonies were isolated to uniform morphology on half-strength yeast extract-tryptone sea salt medium. DNA extraction was carried out using the ZymoBIOMICS DNA miniprep kit, and libraries were prepared using the KAPA Hyper prep kit (Kapa Biosystems, Wilmington, MA). Illumina MiSeq (2 × 150 bp) reads were trimmed using Trimmomatic v0.38 (8), quality was assessed using QUAST (9), and *de novo* assembly was performed using SPAdes v3.12.0 (10). The 16S rRNA genes were amplified using the 27F and 1492R primers (11). Sanger sequencing performed at Genewiz (South Plainfield, NJ) confirmed the purity of the amplicons.

Metagenome sequencing (Illumina NextSeq; 1 × 150 bp) was carried out on enrichment cultures representing six diatom and six dinoflagellate exometabolite treatments. As described previously (5), reads from the six diatom or the six dinoflagellate exometabolite treatments were separately coassembled using MEGAHIT v1.1.3 (12, 13). Default parameters were used for all software unless otherwise specified. Reads were mapped to the assembly with Bowtie v2.2.9 (14) and binned into MAGs using Anvi’o v4 (15, 16) and CONCOCT (17) following published protocols (18) (http://merenlab.org/data/tara-oceans-mags/). The completeness and redundancy of bins were assessed using CheckM v1.0.12 (19). Only bins with >80% completeness and <10% redundancy are reported here (Table 1). The taxonomic classifications of the MAGs were inferred using the Microbial Genome Atlas (MiGA) (http://microbial-genomes.org/) using the TypeMat database (20).

**TABLE 1:** Characteristics of 11 isolate genomes and 38 MAGs obtained from serial enrichments of a coastal bacterial assemblage with metabolites from marine diatoms or dinoflagellates

Isolate or MAG	Taxonomic classification^*a*^	Size (Mb)	G+C content (%)	No. of reads		Coverage (×)		No. of contigs		*N*_50_ (bp)		Compl (%)/cont (%)^*b*^		SRA accession no.	GenBank accession no.	Closest genome (GenBank accession no.)^*c*^	ANI or AAI (%)	
Isolates																		
HF1	Marivivens donghaensis	3.34	60.8	317,066		14.2		24		292,199		99.7/0.8		SRR11481811	JAATOP000000000	*Marivivens* sp. JLT3646 NZ (CP018572)	66.7^*f*^	
HF2	Thalassospira lucentensis	4.50	53.2	477,674		15.9		13		758,710		100.0/0.0		SRR11481803	JAATOV000000000	Thalassospira lucentensis MCCC 1A00383	84.4^*g*^	
HF7	*Pseudooceanicola* sp.	4.27	64.9	312,562		11.0		74		103,803		98.5/0.8		SRR11481802	JAATOW000000000	Pseudooceanicola antarcticus (GCA_002786285)	67.7^*f*^	
HF9A	*Phaeobacter* sp.	4.27	62.2	256,104		9.0		262		30,359		99.3/1.5		SRR11481810	JAATOX000000000	Epibacterium scottomollicae (GCA_003003215)	79.9^*f*^	
HF9B	Vibrio diazotrophicus	4.39	43.3	256,880		8.8		193		47,679		99.8/2.6		SRR11481807	JAATOR000000000	Vibrio diazotrophicus NBRC 103148	91.0^*g*^	
HF15	*Thalassospira* sp.	4.29	54.5	412,760		14.4		25		551,637		100.0/0.0		SRR11481809	JAAVKI000000000	Thalassospira indica NZ (CP031555)	88.0^*g*^	
HF17	Vibrio campbellii	5.66	45.5	273,858		7.3		459		21,883		99.7/1.2		SRR11481808	JAATOQ000000000	Vibrio campbellii NZ (CP019293)	97.8^*g*^	
HF18	Salipiger thiooxidans	5.26	67.5	272,102		7.8		1,007		9,637		95.4/2.8		SRR11481806	JAATOS000000000	Salipiger thiooxidans (GCA_900102075)	97.8^*g*^	
HF31	*Celeribacter* sp.	3.60	59.9	605,892		25.2		13		522,533		99.2/0.5		SRR11481805	JAATOT000000000	Celeribacter ethanolicus NZ (CP022196)	84.5^*g*^	
HF66	*Pseudoalteromonas* sp.	4.39	40.7	692,080		23.6		48		210,054		100.0/0.4		SRR11481804	JAATOU000000000	Pseudoalteromonas shioyasakiensis (GCA_001550135)	91.6^*g*^	
HF70	Vibrio hepatarius	4.71	45.9	382,864		12.2		74		168,419		100.0/0.9		SRR11481812	JAATOO000000000	Vibrio hepatarius (GCA_001274785)	92.6^*g*^	
MAGs^*d*^																	
HF-Dia02	*Oceanospirillaceae*	4.47	42.0	423,160		14.2		283		24,277		86.5/3.0		SRR11451986	JABXIF000000000	Marinomonas polaris DSM 16579	81.6^*g*^	
HF-Dia03	*Rhodobacterales*	3.64	53.4	1,038,613		42.8		201		31,207		95.0/0.8		SRR11451960	JABXHV000000000	Labrenzia suaedae (GCA_900142725)	64.5^*f*^	
HF-Dia04	*Cryomorphaceae*	3.29	45.4	302,680		13.8		182		29,213		97.9/9.7		SRR11451963	JABXHS000000000	Phaeocystidibacter luteus (GCA_008933115)	82.4^*g*^	
HF-Dia05	*Flavobacteriales*	2.53	40.3	1,017,060		60.3		97		147,211		93.1/2.7		SRR11451996	JABXHN000000000	Arenibacter aquaticus (GCA_003957295)	59.6^*f*^	
HF-Dia07	*Alteromonadaceae*	5.10	47.9	16,809,600		494.4		123		84,748		99.9/2.8		SRR11451997	JABXHM000000000	Alteromonas alba (GCA_002993365)	77.4^*f*^	
HF-Dia11	*Flavobacteriales*	2.94	32.3	429,240		21.9		98		76,707		98.3/1.1		SRR11451994	JABXHX000000000	*Tenacibaculum* sp. DSM 106434	65.1^*f*^	
HF-Dia12	*Gammaproteobacteria*	3.42	40.8	517,560		22.7		285		17,653		93.4/2.2		SRR11451988	JABXID000000000	Psychrosphaera saromensis (GCA_002954545)	64.2^*f*^	
HF-Dia14	*Gammaproteobacteria*	3.64	51.8	485,333		20.0		287		19,154		95.1/4.9		SRR11451964	JABXHR000000000	Thiohalobacter thiocyanaticus NZ (AP018052)	46.4^*f*^	
HF-Dia15	*Rhizobiales*	3.97	45.9	304,367		11.5		295		19,983		91.4/3.0		SRR11451989	JABXIC000000000	Terasakiella pusilla DSM 6293 (GCA_000688235)	68.8^*f*^	
HF-Dia17	*Oceanospirillales*	3.38	51.2	22,932,173		1,017.7		418		10,263		91.2/3.3		SRR11451974	JABXHP000000000	Marinobacterium mangrovicola (GCA_004339595)	62.1^*f*^	
HF-Dia18	*Rhodobacteraceae*	4.51	63.1	24,212,687		805.3		409		15,672		93.0/5.3		SRR11451991	JABXIA000000000	Leisingera daeponensis DSM 23529	86.7^*g*^	
HF-Dia21	*Oceanospirillales*	4.53	40.5	1,265,380		41.9		96		99,744		99.2/0.4		SRR11451993	JABXHY000000000	Pleionea mediterranea (GCA_003148745)	62.4^*f*^	
HF-Dia22	*Flavobacteriia*	4.70	40.8	463,733		14.8		179		42,273		98.6/1.4		SRR11451985	JABXHO000000000	“*Candidatus* Fluviicola riflensis” (CP022585)	56.2^*f*^	
HF-Dia23	*Tenacibaculum* sp.	3.20	31.7	424,533		19.9		86		70,967		98.5/0.8		SRR11451962	JABXHT000000000	Tenacibaculum discolor (GCA_003664185)	97.8^*g*^	
HF-Dia27	*Oceanospirillales*	3.64	45.2	2,817,360		116.1		196		31,979		98.3/2.6		SRR11451965	JABXHQ000000000	Pleionea mediterranea (GCA_003148745)	64.1^*f*^	
HF-Dia28^*e*^	Ruegeria pomeroyi	4.51	64.0	60,900,033		2,025.5		200		33,165		97.6/2.2		SRR9668573	JABXIZ000000000	Ruegeria pomeroyi DSS-3	100.0^*g*^	
HF-Dia32	*Flavobacteriales*	3.09	45.8	861,080		41.8		76		69,153		97.7/0.5		SRR11451995	JABXHW000000000	Phaeocystidibacter luteus (GCA_008933115)	67.2^*f*^	
HF-Dia38	*Cyclobacteriaceae*	3.39	40.8	2,743,640		121.4		460		8,688		86.7/4.1		SRR11451992	JABXHZ000000000	Algoriphagus kandeliae (GCA_004571135)	82.1^*g*^	
HF-Dia39	*Marivivens* sp.	2.49	56.7	7,811,960		470.6		142		23,748		95.1/1.0		SRR11451961	JABXHU000000000	*Marivivens* sp. JLT3646 NZ (CP018572)	97.2^*g*^	
HF-Dia40	*Vibrionaceae*	4.24	46.3	7,453,920		263.7		357		16,672		96.6/2.1		SRR11451990	JABXIB000000000	Vibrio hepatarius (GCA_001274785)	92.6^*g*^	
HF-Din01	*Salipiger* sp.	4.40	68.0	10,301,867		351.2		701		7,265		86.8/2.7		SRR11451976	JABXIO000000000	*Salipiger thiooxidans* (GCA_900102075)	98.0^*g*^	
HF-Din02	*Methylocystaceae*	4.09	48.4	13,224,333		485.0		69		276,225		100.0/1.0		SRR11451984	JABXIG000000000	Terasakiella pusilla DSM 6293	79.0^*f*^	
HF-Din03^*e*^	Ruegeria pomeroyi	4.54	64.0	36,062,733		1,191.5		78		105,484		98.7/0.7		SRR9668574	JABXIY000000000	Ruegeria pomeroyi DSS-3	100.0^*g*^	
HF-Din05	*Gammaproteobacteria*	3.45	45.4	425,500		18.5		223		24,639		93.7/2.2		SRR11451969	JABXIU000000000	Pseudoalteromonas phenolica O-BC30 GCA 004103265	48.7^*f*^	
HF-Din08	*Oceanospirillaceae*	5.78	58.6	3,787,827		98.3		249		42,217		98.1/2.0		SRR11451978	JABXIM000000000	Marinobacterium rhizophilum DSM 18822	68.5^*g*^	
HF-Din09	*Flammeovirgaceae*	4.04	42.1	2,308,187		85.7		543		9,559		92.8/2.7		SRR11451975	JABXIP000000000	Roseivirga spongicola (GCA_004103265)	81.4^*f*^	
HF-Din10	*Alphaproteobacteria*	3.00	54.3	1,760,000		88.0		309		14,274		81.9/1.7		SRR11451968	JABXIV000000000	*Hankyongella ginsenosidimutans* W1-2-3 (CP039704)	50.2^*f*^	
HF-Din11	Vibrio campbellii	5.63	45.3	36,542,453		973.6		92		123,020		100.0/0.1		SRR11451983	JABXIH000000000	Vibrio campbellii NZ (CP019293)	97.9^*g*^	
HF-Din12	*Oceanospirillales*	2.58	41.6	1,683,880		97.9		62		69,368		98.1/4.1		SRR11451973	JABXIQ000000000	Kangiella profundi NZ (CP025120)	63.5^*f*^	
HF-Din13	*Campylobacteraceae*	2.76	28.4	1,264,080		68.7		38		142,008		99.2/1.2		SRR11451982	JABXII000000000	Halarcobacter bivalviorum strain LMG 26154 (CP031217)	85.2^*g*^	
HF-Din14	*Rhodobacteraceae*	3.53	59.9	13,734,053		583.6		64		109,963		98.9/0.5		SRR11451979	JABXIL000000000	Celeribacter halophilus (GCA_003254175)	82.0^*g*^	
HF-Din16	*Flavobacteriia*	1.81	37.0	97,740		8.1		234		9,657		83.0/2.3		SRR11451970	JABXIT000000000	Maribacter cobaltidurans NZ (CP022957)	57.8^*f*^	
HF-Din17	*Gammaproteobacteria*	4.03	44.2	354,640		13.2		487		12,701		85.8/4.3		SRR11451967	JABXIW000000000	*Pleionea mediterranea* (GCA_003148745)	49.3^*f*^	
HF-Din19	*Flavobacteriales*	2.37	29.8	260,700		16.5		69		52,100		97.9/1.4		SRR11451981	JABXIJ000000000	Polaribacter porphyrae (GCA_002954685)	68.7^*f*^	
HF-Din21	*Kordiimonadaceae*	3.62	53.6	1,908,947		79.1		96		81,569		97.1/1.8		SRR11451971	JABXIS000000000	Kordiimonas lipolytica (GCA_001550065)	76.8^*f*^	
HF-Din22	*Cyclobacteriaceae*	3.65	40.7	1,498,933		61.6		38		178,308		99.6/0.6		SRR11451977	JABXIN000000000	Algoriphagus kandeliae (GCA_004571135)	81.1^*g*^	
HF-Din24	*Gammaproteobacteria*	3.50	44.0	22,920,333		982.3		151		35,787		98.5/2.3		SRR11451980	JABXIK000000000	Oceanobacter kriegii DSM 6294 (GCA_000422845)	54.6^*f*^	
HF-Din29	*Gammaproteobacteria*	2.11	49.9	753,973		53.6		305		8,218		82.8/2.9		SRR11451972	JABXIR000000000	Spongiibacter marinus DSM 19753 (GCA_000422345)	51.2^*f*^	

a Taxonomic classification of isolates is based on >98% similarity of 16S rRNA genes or >98% similarity of *hsp60* genes (*Vibrio* only). Taxonomic classification of metagenome-assembled genomes (MAGs) is based on the lowest taxonomic level with a *P* value of ≤0.015 as calculated in MiGA using the TypeMat database, in which average nucleotide identity (ANI) is generally reported for similarities of ≥85%, and average amino acid identity (AAI) is reported if the ANI is <85%.

b Compl, completeness level; cont, contamination level.

c Closest genome was determined by MiGA analysis using the TypeMat database.

d MAGs that start with HF-Dia are assembled from the six diatom metagenomes, while MAGs that start with HF-Din are assembled from the six dinoflagellate metagenomes.

e Previously published (5).

f AAI.

g ANI.

### Data availability.

All data are deposited under GenBank BioProject number PRJNA553557. The raw reads of the genomic data for the isolates are deposited under SRA accession numbers SRR11481802 to SRR11481812. The original raw reads of the metagenomic data used for MAG assembly are deposited under SRA accession numbers SRR11434620 to SRR11434631. The assemblies for isolates and MAGs are deposited under the GenBank accession numbers listed in Table 1; the versions described in this paper are the first versions.

## References

[1] Amin SA, Parker MS, Armbrust EV 2012 Interactions between diatoms and bacteria. Microbiol Mol Biol Rev 76:667–684. doi:10.1128/MMBR.00007-12. 22933565 PMC3429620

[2] Azam F, Fenchel T, Field JG, Gray JS, Meyer-Reil LA, Thingstad F 1983 The ecological role of water-column microbes in the sea. Mar Ecol Prog Ser 10:257–263. doi:10.3354/meps010257.

[3] Azam F, Malfatti F 2007 Microbial structuring of marine ecosystems. Nat Rev Microbiol 5:782–791. doi:10.1038/nrmicro1747. 17853906

[4] Seymour JR, Amin SA, Raina J-B, Stocker R 2017 Zooming in on the phycosphere: the ecological interface for phytoplankton–bacteria relationships. Nat Microbiol 2:17065. doi:10.1038/nmicrobiol.2017.65. 28555622

[5] Fu H, Uchimiya M, Gore J, Moran MA 2020 Ecological drivers of bacterial community assembly in synthetic phycospheres. Proc Natl Acad Sci U S A 117:3656–3662. doi:10.1073/pnas.1917265117. 32015111 PMC7035482

[6] Amin SA, Hmelo LR, van Tol HM, Durham BP, Carlson LT, Heal KR, Morales RL, Berthiaume CT, Parker MS, Djunaedi B, Ingalls AE, Parsek MR, Moran MA, Armbrust EV 2015 Interaction and signalling between a cosmopolitan phytoplankton and associated bacteria. Nature 522:98–101. doi:10.1038/nature14488. 26017307

[7] Landa M, Burns AS, Roth SJ, Moran MA 2017 Bacterial transcriptome remodeling during sequential co-culture with a marine dinoflagellate and diatom. ISME J 11:2677–2690. doi:10.1038/ismej.2017.117. 28731474 PMC5702724

[8] Bolger AM, Lohse M, Usadel B 2014 Trimmomatic: a flexible trimmer for Illumina sequence data. Bioinformatics 30:2114–2120. doi:10.1093/bioinformatics/btu170. 24695404 PMC4103590

[9] Gurevich A, Saveliev V, Vyahhi N, Tesler G 2013 QUAST: quality assessment tool for genome assemblies. Bioinformatics 29:1072–1075. doi:10.1093/bioinformatics/btt086. 23422339 PMC3624806

[10] Bankevich A, Nurk S, Antipov D, Gurevich AA, Dvorkin M, Kulikov AS, Lesin VM, Nikolenko SI, Pham S, Prjibelski AD, Pyshkin AV, Sirotkin AV, Vyahhi N, Tesler G, Alekseyev MA, Pevzner PA 2012 SPAdes: a new genome assembly algorithm and its applications to single-cell sequencing. J Comput Biol 19:455–477. doi:10.1089/cmb.2012.0021. 22506599 PMC3342519

[11] Frank JA, Reich CI, Sharma S, Weisbaum JS, Wilson BA, Olsen GJ 2008 Critical evaluation of two primers commonly used for amplification of bacterial 16S rRNA genes. Appl Environ Microbiol 74:2461–2470. doi:10.1128/AEM.02272-07. 18296538 PMC2293150

[12] Li D, Liu CM, Luo R, Sadakane K, Lam TW 2015 MEGAHIT: an ultra-fast single-node solution for large and complex metagenomics assembly via succinct de Bruijn graph. Bioinformatics 31:1674–1676. doi:10.1093/bioinformatics/btv033. 25609793

[13] Li D, Luo R, Liu CM, Leung CM, Ting HF, Sadakane K, Yamashita H, Lam TW 2016 MEGAHIT v1.0: a fast and scalable metagenome assembler driven by advanced methodologies and community practices. Methods 102:3–11. doi:10.1016/j.ymeth.2016.02.020. 27012178

[14] Langmead B, Salzberg S 2012 Fast gapped-read alignment with Bowtie 2. Nat Methods 9:357–359. doi:10.1038/nmeth.1923. 22388286 PMC3322381

[15] Eren AM, Esen ÖC, Quince C, Vineis JH, Morrison HG, Sogin ML, Delmont TO 2015 Anvi’o: an advanced analysis and visualization platform for ’omics data. PeerJ 3:e1319. doi:10.7717/peerj.1319. 26500826 PMC4614810

[16] Delmont TO, Eren AM 2016 Identifying contamination with advanced visualization and analysis practices: metagenomic approaches for eukaryotic genome assemblies. PeerJ 4:e1839. doi:10.7717/peerj.1839. 27069789 PMC4824900

[17] Alneberg J, Bjarnason BS, De Bruijn I, Schirmer M, Quick J, Ijaz UZ, Lahti L, Loman NJ, Andersson AF, Quince C 2014 Binning metagenomic contigs by coverage and composition. Nat Methods 11:1144–1146. doi:10.1038/nmeth.3103. 25218180

[18] Delmont TO, Quince C, Shaiber A, Esen ÖC, Lee ST, Rappé MS, McLellan SL, Lücker S, Eren AM 2018 Nitrogen-fixing populations of Planctomycetes and Proteobacteria are abundant in surface ocean metagenomes. Nat Microbiol 3:804–813. doi:10.1038/s41564-018-0176-9. 29891866 PMC6792437

[19] Parks DH, Imelfort M, Skennerton CT, Hugenholtz P, Tyson GW 2015 CheckM: assessing the quality of microbial genomes recovered from isolates, single cells, and metagenomes. Genome Res 25:1043–1055. doi:10.1101/gr.186072.114. 25977477 PMC4484387

[20] Rodriguez-R LM, Gunturu S, Harvey WT, Rosselló-Mora R, Tiedje JM, Cole JR, Konstantinidis KT 2018 The Microbial Genomes Atlas (MiGA) webserver: taxonomic and gene diversity analysis of Archaea and Bacteria at the whole genome level. Nucleic Acids Res 46:W282–W288. doi:10.1093/nar/gky467. 29905870 PMC6031002

